# DNA microarray analysis of *Salmonella *serotype Typhimurium strains causing different symptoms of disease

**DOI:** 10.1186/1471-2180-10-96

**Published:** 2010-03-31

**Authors:** Eva Litrup, Mia Torpdahl, Burkhard Malorny, Stephan Huehn, Morten Helms, Henrik Christensen, Eva M Nielsen

**Affiliations:** 1 Statens Serum Institut, Bacterial Typing, Artillerivej 5, DK-2300 Copenhagen S, Denmark; 2 Federal Institute for Risk Assessment, National Reference Laboratory for Salmonella, Diedersdorfer Weg 1, D-12277 Berlin, Germany; 3 Department of Internal Medicine, Roskilde Sygehus, 4000 Roskilde, Denmark; 4 University of Copenhagen, Faculty of Life Sciences, Bülowsvej 17, DK-1870 Frederiksberg C, Denmark

## Abstract

**Background:**

*Salmonella enterica *subsp. *enterica *is one of the leading food-borne pathogens in the USA and European countries. Outcome of human *Salmonella *serotype Typhimurium infections ranges from mild self-limiting diarrhoea to severe diarrhoea that requires hospitalization. Increased knowledge of the mechanisms that are responsible for causing infection and especially the severity of infection is of high interest.

**Results:**

Strains were selected from patients with mild infections (n = 9) and patients with severe infections (n = 9) and clinical data allowed us to correct for known underlying diseases. Additionally, outbreak isolates (n = 3) were selected. Strains were analyzed on a DNA-DNA microarray for presence or absence of 281 genes covering marker groups of genes related to pathogenicity, phages, antimicrobial resistance, fimbriae, mobility, serotype and metabolism. Strains showed highly similar profiles when comparing virulence associated genes, but differences between strains were detected in the prophage marker group. The *Salmonella *virulence plasmid was present in 72% of the strains, but presence or absence of the virulence plasmid did not correspond to disease symptoms. A dendrogram clustered strains into four groups. Clustering confirmed DT104 as being a clonal phagetype. Clustering of the remaining strains was mainly correlated to presence or absence of the virulence plasmid and mobile elements such as transposons. Each of the four clusters in the tree represented an almost equal amount of strains causing severe or mild symptoms of infection.

**Conclusions:**

We investigated clinical significance of known virulence factors of *Salmonella *serotype Typhimurium strains causing different disease symptoms, and conclude that the few detected differences in *Salmonella *serotype Typhimurium do not affect outcome of human disease.

## Background

Globally, *Salmonella enterica *subsp. *enterica *is one of the leading food-borne pathogens. For example in 2006 in the United States, *Salmonella enterica *subsp. *enterica *caused 45.808 registered cases of salmonellosis, corresponding to an incidence of 15 cases/100,000 inhabitants [1]. Furthermore the actual number of infections is estimated to be 38 times higher [2]. In Denmark, there were 1658 registered cases of salmonellosis (incidence of 30 cases/100,000 inhabitants) in 2006 [3]. *Salmonella *serotype Typhimurium, denoted *S*. Typhimurium, accounted for 17% of the salmonellosis cases in the USA and 25% of the Danish cases [1,3]. The outcome of human infection ranges from mild self-limiting diarrhoea to severe diarrhoea that requires hospitalization. In rare cases, often among immunocompromised patients, salmonellosis can be fatal.

Several factors in both the host and the bacteria influence the outcome of an infection. Clearly an important aspect of human infection is the immune state of the patient. It has been shown that immunocompromised patients are more prone to develop a severe infection [4]. Another important aspect of human infection is the intestinal microbiota of the host. Ingestion of antibiotics is known to affect the intestinal microbiota leaving the host more prone to infection and disease caused by *S*. Typhimurium [5].

Significant bacterial factors for the outcome of infection are encoded by a wide range of genetic elements, including plasmids, prophages and *Salmonella *Pathogenicity Islands (SPIs). A total of 14 SPIs have been described so far [6]. SPI-1 encodes type 3 secretion system 1 (T3SS-1) that causes secretion and translocation of a range of bacterial proteins to the host cell. SPI-2 encodes T3SS-2 that allows intracellular survival and replication [7].

Different *S*. Typhimurium strains share more than 99% genomic content [8]. The detected variation within *S*. Typhimurium is primarily represented by the prophages in the genome [9]. Variation in phenotype has also been demonstrated as there are different phagetypes of *S*. Typhimurium strains, and some of them can even show a high degree of variation in host adaptation [10]. Intra-serotype variability is also caused by the plasmids carried by *S*. Typhimurium, in particular, the *Salmonella *Virulence Plasmid (pSLT) which was observed more frequently in the strains isolated from blood than the strains isolated from faeces [11]. It has been proposed that this plasmid is significant in the spreading of an infectious strain from the intestine [12].

The recent development of microarray technology has allowed an extensive screening of many *S*. Typhimurium strains [13-15], but to our knowledge, no study has been able to link the molecular data obtained by microarray analysis of the strains to detailed epidemiological and clinical patient data.

We analyzed a collection of *S*. Typhimurium strains by DNA microarray analysis. These strains were selected on the basis of a previous epidemiological study where clinical data were obtained by means of patient interviews. The strains were selected from patients with mild infections and from patients with severe infections, and clinical data allowed us to correct for known underlying diseases and patient age. Strains were analyzed for presence or absence of 281 genes covering marker groups of genes related to pathogenicity, phages, antimicrobial resistance, fimbriae, mobility, serotype, and metabolism. We show that *S*. Typhimurium strains causing very different symptoms in patients had little genomic variation, and the observed variation does not correlate to the severity of disease.

## Results

### Subtyping

All strains were subtyped by Pulsed-field gel electrophoresis (PFGE), Multiple-locus variable-number of tandem-repeat analysis (MLVA) and Multilocus sequence typing (MLST). In general, the PFGE types of the strains correspond to the phagetype. All of the phagetype DT12 strains had the PFGE 22 profile and five out of six DT104 strains had the PFGE 14 profile. The remaining phagetypes showed different PFGE profiles (see additional file 1: *Xba *I PFGE profiles of all isolates).

The MLVA types of the strains were all different. Loci STTR-9 and STTR-3 were the most conserved alleles and they had 1, 2 or 3 repeat units. STTR-5, STTR-6 and STTR-10 were all alleles with varying repeat units. Some strains did not contain the STTR-10 allele at all, corresponding well to the fact that these strains were not carrying the pSLT (see additional file 2: Typing results of all strains).

The Sequence types (ST) of the strains were primarily ST19. Only three strains had other STs and these were ST376, ST35 and ST34 (see additional file 2: Typing results of all strains).

### DNA microarray marker groups Resistance and Serotyping

The DNA microarray included 49 probes that targeted 10 different resistance genes and some of their known variants. The phenotypic resistance profiles all corresponded to the results obtained by the array (see additional file 3: Microarray results of all markers). The DNA microarray included 33 probes that target different O-serogroups and H-antigens. The *S*. Typhimurium serotype was confirmed by the array (see additional file 3: Microarray results of all markers).

### DNA microarray marker groups Metabolism, Prophage and Mobility

The DNA microarray contained 20 probes targeting different genes in the metabolism marker group. All strains on the array possessed 18 of the 20 metabolism genes. The gene *SEN4287*, which has been observed in serotypes *S*. Enteritidis, *S*. Dublin and *S*. Gallinarum and the gene *STY4221*, which has been found in serotypes *S*. Typhi and *S*. Paratyphi A, were not detected in any of the *S*. Typhimurium strains.

The DNA microarray contained 10 probes targeting different genes in the prophage marker group. Prophage related genes showed different present/absent profiles in different strains. All DT104 strains displayed identical profiles within the prophage marker group. Only DT104 strains possessed the ORF84 marker. The prophage genes *sb10 *and *sb54 *were present in all DT104 strains and some other strains. The genes *STY3672*, *STY3676 *and *STY4625 *were present in both DT10 strains and one of three RDNC strains. The remaining genes in the prophage marker group were identically present/absent in all strains (see additional file 3: Microarray results of all markers).

The DNA microarray contained 57 probes targeting different genes in the mobility marker group which also included plasmid incompatibility markers and IS-element markers. The markers showed high variability in the present/absent pattern among all strains. The variability correlated well with known properties of strains, e.g. all strains lacking the pSLT, also lacked the gene encoding the typical FIIA replicon of pSLT, and these strains also lacked the *traT *gene that is encoded in pSLT (see additional file 3: Microarray results of all markers).

### DNA microarray marker groups Pathogenicity and Fimbrial

The pathogenicity marker group consisted of 87 markers and the strains had a highly similar pattern of present/absent genes within this marker group. Only the DT104 strains showed variation, as they lacked the *gipA *gene encoded by the Gifsy-1 prophage but harboured the two other genes encoded by Gifsy-1. The DT104 strains also possessed two other prophage-related genes which no other strains possessed. All strains harboured prophage Gifsy-2 and lacked prophages Gifsy-3 and Fels-1. Five strains of five different phagetypes lacked the pSLT and, therefore, lacked the virulence genes encoded in the plasmid. In total, 72% of the strains in this study carried the pSLT. The same percentage is observed when comparing all of the *S*. Typhimurium strains detected in Denmark between 2005 and 2009 (data not shown).

**Figure 1:**
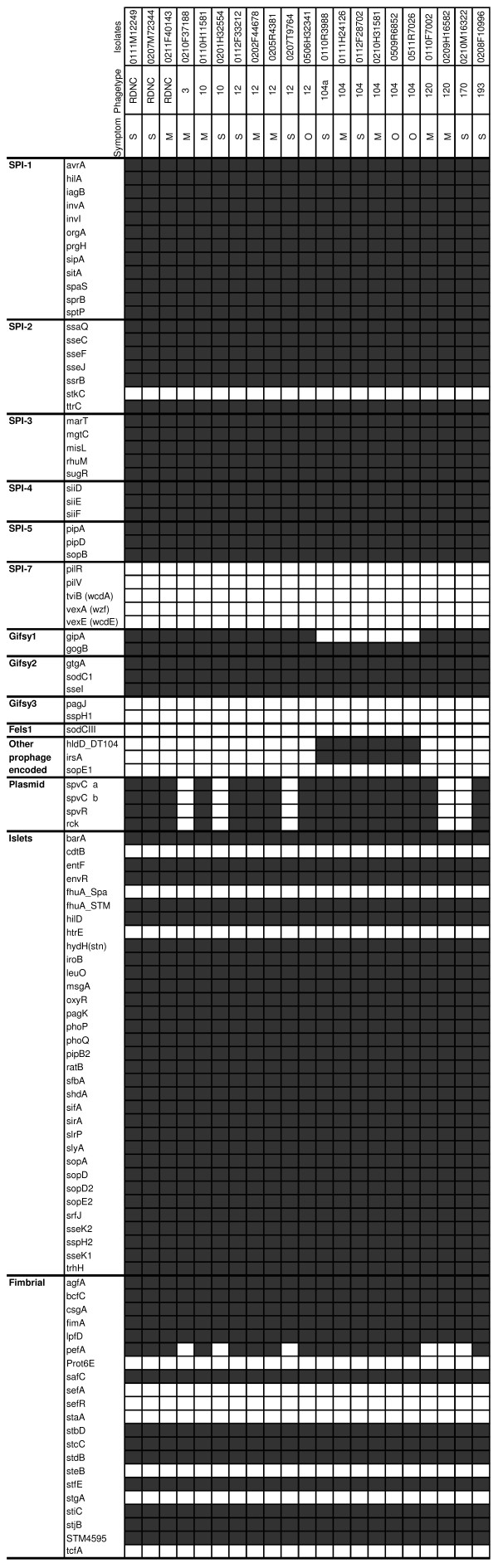
**Microarray results of virulence associated markers**. Markers are listed according to genomic location or function. A grey box indicates that the marker is present, and a white box indicates that the marker is absent.

The SPI-1 to SPI-5 were present and SPI-7 was absent in all strains (Fig. 1).

The DNA microarray contained 22 probes targeting different genes in the fimbrial marker group. All strains showed identical patterns within this marker group, except for the *pefA *gene which is encoded in the pSLT. One strain carrying the pSLT did not show a positive reaction in the *pefA *probe (Fig. 1).

### Clustering of strains

**Figure 2:**
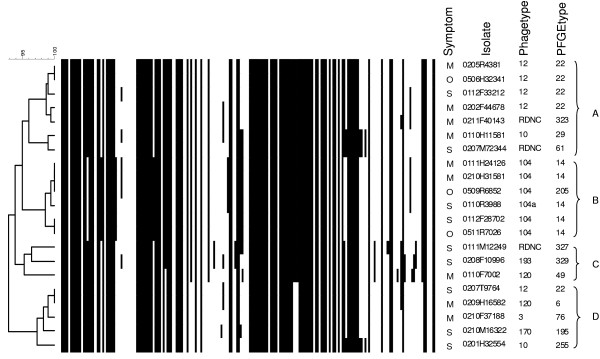
**UPGMA dendrogram**. UPGMA dendrogram calculated on microarray results as binary coefficients by simple matching, markers for serotype and resistance are not included. Each marker is listed along the horizontal top of the dendrogram, and a black line in the figure represents a positive hybridisation and thus gene present. Four clusters indicated by letters A-D. M = Mild symptoms, S = Severe symptoms, O = Outbreak.

The microarray analysis clustered the strains into four major branches in a dendrogram (Fig. 2). The dendrogram is calculated from all markers except the resistance and serotyping markers as these could create a bias in the analysis. Cluster A had a depth of 96.1% and contained most of the DT12 strains but also other phagetypes. The strains in cluster A all harboured the pSLT, and all seven strains were fully sensitive to antimicrobial agents (see additional file 2: Typing results of all strains). In cluster A, two strains represented severe infection, four strains represented mild infection, and there was one outbreak strain. Cluster B had a depth of 98.6% and contained all six DT104 strains, which all harboured the pSLT. Two of the DT104 strains were fully susceptible to antimicrobial agents. In cluster B, two strains represented severe infection, two strains represented mild infection, and additionally there were two outbreak strains.

Cluster C had a depth of 95.2% and contained only three strains of three different phagetypes. All of the three strains carried the pSLT and showed resistance to at least four antimicrobial agents. The strains in cluster C branch off separately as they possess more genes from the mobility marker group which includes transposases. In cluster C, two strains represented severe infection and one strain represented mild infection. Cluster D had a depth of 97.2% and contained five strains of different phagetypes, including a DT12 strain, but none of the strains harboured the pSLT. One strain in cluster D showed resistance to three antimicrobial agents. In cluster D, three strains represented severe infection while two strains represented mild infection. In conclusion, strains causing severe and mild infection were represented equally across the dendrogram (Fig. 2).

## Discussion

A collection of *S*. Typhimurium strains were analyzed and compared by the use of a microarray designed for characterization of *Salmonella*. The array consisted of 281 markers covering marker groups of genes related to pathogenicity, phages, antimicrobial resistance, fimbriae, mobility, serotype, and metabolism. Extensive background knowledge of patients regarding symptoms and underlying diseases enabled us to compare strains causing mild symptoms to strains causing severe symptoms.

Initial subtyping of the strains was performed using MLST and MLVA. These two methods target different parts of the chromosome and areas with different genetic variability, leading to differences in the discriminatory power of the methods. MLVA methods are generally high-discriminatory typing methods developed for outbreak investigations, and the *S*. Typhimurium MLVA method used in this study was specifically developed to differentiate the highly similar DT104 clone [16]. This method is based on highly variable repeat regions on the chromosome and on a plasmid. MLST is developed to estimate long term development and is based on conserved housekeeping genes with minimal variation [17]. Most strains in this study belonged to ST19, except for three strains with different STs. The difference between ST19 and each of the other STs was a single nucleotide change in one allele, so the similarity of the strains was high as expected with MLST. The strains all had different MLVA profiles, corresponding well with the fact that MLVA is a more discriminatory typing method and the strains were selected to be epidemiologically unrelated.

The strains were tested for antimicrobial resistance and eight of 21 strains showed resistance to three or more antimicrobial agents. Three strains from the group with mild symptoms, four strains from the group with severe symptoms, and a single outbreak strain showed resistance to antimicrobial agents. The resistance pattern did not correlate with the severity of disease in patients. The lack of increased virulence of resistant strains has previously been shown in DT104 strains [18,19]. An American study described that humans who ingested antimicrobials are more prone to get a subsequent infection with a resistant *S*. Typhimurium strain [20]. Two of the patients included in our study were administered antimicrobials within a month before onset of the *S*. Typhimurium infection, one patient was in the group with severe symptoms and the other patient was in the group with mild symptoms. Both infections were caused by resistant *S*. Typhimurium strains.

Differences between the *S*. Typhimurium strains were detected in the prophage marker group. The DT104 strains were different as seen by detection of the ORF84 marker, previously shown to be present primarily in DT104 strains [21]. The *Salmonella *prophage ST64B (*sb10 *and *sb54*) was detected in different phagetypes in this study, but notably this prophage is present in all DT104 strains, and these observations corresponded to previous findings [22]. Other genes showing variability within the prophage marker group were the *S*. Typhi specific genes *STY3672*, *STY3676 *and *STY4625*. The markers of these genes were detected in three *S*. Typhimurium strains. The occurrence of these genes could be explained by lacking specificity of the probe, or by point mutations in the *S*. Typhimurium strains leading to cross-hybridization. Prophages are known to contribute to virulence in mice [23] but presence or absence of prophages does not correlate with any differences in symptoms caused by strains in our study investigating strains isolated from humans.

The mobility marker group also displayed variation between strains, but most variation related to incompatibility groups of plasmids and probes encoding transposons. The variation did not correspond to any phagetypes or disease symptoms.

The strains showed highly similar profiles when comparing the virulence associated genes. Some variation was detected between other phagetypes and the DT104 strains which were the only strains containing the *hldD *gene and the *irsA *gene, but these genes have previously been shown to be specific for the DT104 phagetype [24]. Also the Gifsy-1 encoded genes showed variation between other phagetypes and the DT104 strains, as the DT104 strains lacked one of three Gifsy-1 encoded genes present on the array. The gene lacking in our DT104 strains is consistent with an observation made recently in a study comparing the genome sequence of a DT104 strain to a *S*. Typhimurium LT2 strain [25]. The study observes a prophage sequence in DT104 which only shows partly homology to the Gifsy-1 prophage sequence. All other strains in our study possessed the Gifsy-1 prophage. The SPI-1 to SPI-5 were present in all strains but the SPI-7 was absent. SPI-7 was initially reported in a *S*. Typhi [26], and similar islands were detected in *S*. Dublin and *S*. Paratyphi C [27]. The pSLT is another important virulence marker. In an American study, pSLT was shown to be present in 76% of strains isolated from blood compared to 42% of strains isolated from faeces [11], however, in the present study the virulence plasmid was present in 72% of the strains, even though the strains were all isolated from faeces and some strains caused very mild disease symptoms. The selected *S*. Typhimurium strains are representative for the Danish *S*. Typhimurium population regarding the presence of pSLT, as 72% of all Danish *S*. Typhimurium isolates from 2005 until 2009 carried the plasmid. Out of five strains lacking the pSLT, three had caused severe symptoms. Interestingly, strains can cause infection with severe symptoms even if they lack the plasmid. Furthermore, strains can carry the pSLT and only cause infection with mild symptoms. In this study, the presence or absence of pSLT did not correspond to any phagetypes or disease symptoms.

The dendrogram calculated on the basis of the array results showed clustering of the strains into four groups. The clustering confirmed DT104 as being a clonal phagetype, but a number of probes were also designed to target only DT104 strains, and that might emphasize the separate clustering of this phagetype. The clustering of the remaining groups was mainly correlated to the presence or absence of the pSLT and other mobile elements such as transposons. The four clusters in the tree represented an almost equal amount of strains causing severe or mild symptoms of *S*. Typhimurium infections.

The probes on the array were designed primarily on basis of the *S*. Typhimurium LT2 sequence, but also some additional known genes from other serotypes such as *S*. Enteritidis and *S*. Typhi. The presence or absence of additional *S*. Typhimurium genes, which are not present in the LT2 sequence, could not be assessed in this study. It is possible that the presence or absence of such genes, not present in LT2, are responsible for the observed differences in the patient symptoms. Although this is not likely, as recent publications of sequenced *S*. Typhimurium strains showed few gene differences to the LT2 sequenced strain [28,29].

## Conclusion

We investigated a collection of *Salmonella *strains for the presence of a wide range of known virulence genes, and detected no significant difference in the presence of these genes. The investigated strains were carefully selected, based on epidemiological data, to represent strains causing severe symptoms of disease and strains causing mild symptoms of disease. Although the investigated strains had different genomic contents, this study found no evidence of a correlation between the genomic contents of the *S*. Typhimurium strains and the symptoms they caused in human cases of salmonellosis. Based on the results of this study, an idea which immediately suggests itself is that the factors and defence mechanisms of the host immune system may play a fundamental role in the different outcomes of infection.

## Methods

### Patient interviews

Data for the present study was obtained from a prospective cohort study carried out in Denmark from September 2001 to December 2002 [30]. Cases were patients with a culture-confirmed *S*. Typhimurium infection, identified by the examination of samples submitted to Statens Serum Institut (SSI) from hospitals and general practitioners. Patients were invited to participate by their own physicians or the relevant hospital department. Individuals who agreed to participate were mailed a questionnaire and asked to complete the questionnaire immediately. Data was collected by a computer-assisted telephone interviewing system (CATI) whilst the subjects were looking at their questionnaire. This method facilitated data collection and allowed standardized probing about relevant exposures and outcomes. Data collected included information on clinical symptoms, treatment, medications (including antimicrobials) from one month before infection to one month after, underlying illnesses, foreign travel during the two weeks prior to inclusion and basic socioeconomic variables i.e. education, occupation and household income. Cases were excluded for the following reasons: an earlier history of *Salmonella *infection within six month prior to onset of the current infection, lack of communication skills in Danish, or if the patient's health care provider deemed the patient unable to complete interview due to an impaired mental state. A parent was interviewed if the patient was under 15 years of age and if the patients were between 15 and 18 years of age they could be interviewed - subject to parental approval. This study was reported to The Danish Data Protection Agency and has been approved by the regional scientific ethical committee of Copenhagen and Frederiksberg Municipality (KEF 01-031/01).

### Selection of strains

Faecal strains of *S*. Typhimurium were chosen based on the previously described patient interviews. Strains from patients over the age of 65 years and strains from patients with known underlying diseases were not included in this study. The patients were sorted according to hospitalization data and fever. Two groups were then established: a severe infection group with patients who were hospitalized due to their *S*. Typhimurium infection and also had a fever; and a mild infection group with patients who were not hospitalized and did not have a fever. From each of these groups nine strains were selected, aiming to represent the same phagetypes in each group (Table 1). The phagetype distribution observed within all strains from the interviewed patients, not including the patients with known underlying disease, correlated to the overall distribution of all human *S*. Typhimurium strains in Denmark in 2001 and 2002. A pattern of specific phagetypes relating to specific symptoms was not observed within the entire interview material (data not shown). Of the nine hospitalized patients, four had bloody stools. Furthermore, three outbreak strains were included in the study, representing strains with known high virulence potential. All faecal samples received at SSI in Denmark are screened for double infection with frequently occurring intestinal pathogens such as *Campylobacter*, *Shigella*, *Yersinia *and others. All strains used in this study were confirmed as originating from a single-organism infection.

**Table 1:** Isolate information and degree of disease symptoms.

Isolate nr	Phage type	Patient age	Year of isolation	Disease symptoms
0210F37188	3	39	2002	Mild
0110H11581	10	38	2001	Mild
0202F44678	12	30	2002	Mild
0205R4381	12	41	2002	Mild
0111H24126	104	62	2001	Mild
0210H31581	104	14	2002	Mild
0110F7002	120	63	2001	Mild
0209H16582	120	0	2002	Mild
0211F40143	RDNC	1	2002	Mild
0201H32554	10	3	2002	Severe
0112F33212	12	47	2001	Severe
0207T9764	12	11	2002	Severe
0112F28702	104	20	2001	Severe
0110R3988	104a	36	2001	Severe
0210M16322	170	19	2002	Severe
0208F10996	193	9	2002	Severe
0111M12249	RDNC	12	2001	Severe
0207M72344	RDNC	16	2002	Severe
0506H32341	12	53	2005	Outbreak
0509R6852	104	58	2005	Outbreak
0511R7026	104	2	2005	Outbreak

### Serotyping

All strains were previously serotyped at SSI according to the White-Kauffmann-Le Minor scheme [31] by agglutination with O- and H-antigen specific sera (SSI Diagnostika, Hillerød, Denmark). All strains belonged to *Salmonella enterica *serotype Typhimurium.

### Antimicrobial susceptibility testing

Susceptibility to a standard panel of antimicrobial agents http://www.danmap.org was determined by microbroth dilution and interpreted according to Clinical and Laboratory Standards Institute guidelines [32], except for ciprofloxacin (≥0.125 μg/mL was used as breakpoint).

### Phage typing

Phage typing was performed by the National Food Institute, Technical University of Denmark according to the phage typing scheme developed by Callow [33] and extended by Anderson et al. [34]. Strains that react with phages, but have a profile not included in the phage typing scheme, are denoted Reaction Does Not Conform (RDNC).

### DNA microarray

The DNA microarray used in this study was previously described [35]. A set of 281 gene-specific 57-60 mer oligonucleotide probes were designed using the program Array Designer 4.1 (Premier Biosoft, Palo Alto, CA, USA). The oligonucleotides were spotted on glass slides using a QArray Mini Arrayer (Genetix, New Milton, UK). Hybridized spots were visualized in a GenePix 4000B laser scanner (Axon, Foster City, CA). Each oligonucleotide allows the detection of the presence or absence of a characteristic sequence previously described in *Salmonella*. The microarray used gives no information on the location of a gene or target sequence and can only score its presence or absence. Uncertain array results were resolved by PCR using primers described previously [35].

### Data analysis

Analysis of the DNA microarray data was performed as previously described [35]. A comparison was made by importing array values, gene present or absent, into BioNumerics 5.1 (Applied Maths, Sint-Martens-Latem, Belgium) as character data. An Unweighted Pair Group Method with Arithmetic mean (UPGMA) dendrogram (Fig. 2) was calculated by simple matching of binary coefficients on the basis of a geneset consisting of all genes from the array except the serotyping markers and the resistance markers.

### Multiple-locus variable-number of tandem-repeat analysis (MLVA)

MLVA was performed as described previously [36] to ensure that the strains were likely to be epidemiologically unrelated. The MLVA repeats were calculated and named according to the method described recently [37].

### Pulsed-field gel electrophoresis (PFGE)

PFGE was carried out with *Xba*I restriction enzyme according to the Pulse-Net protocol [38], gels were analyzed in BioNumerics 5.1, and profiles were assigned by comparison to a database with known Danish profiles (see additional file 1: *Xba *I PFGE profiles of all isolates).

### Sequence typing

Multilocus sequence typing (MLST) was carried out as previously described [39] and the alleles and the sequence types were assigned according to the MLST scheme on http://mlst.ucc.ie/mlst/mlst/dbs/Senterica/.

## Authors' contributions

EL carried out the microarray experiments, the MLST analysis, participated in the study design and drafted the manuscript. MT participated in conceiving and designing the study. BM designed the microarray. SH participated in the microarray experiments and participated in drafting the Methods section. MH carried out the patient interviews and the epidemiological analysis and participated in drafting the Methods section. HC participated in conceiving and designing the study. EMN participated in conceiving and designing the study. All authors read and approved the final manuscript.

## Supplementary Material

Additional file 1 **PFGE profiles**. *Xba *I PFGE profiles of all isolates Click here for file

Additional file 2 Typing results of all strains Click here for file

Additional file 3 **Microarray results of all markers**. Markers are listed alphabetically within marker groups. A grey box indicates the marker being present and a white box indicates the marker being absent. Click here for file
