# Supplementary figures and images for: DNA microarray analysis of Salmonella serotype Typhimurium strains causing different symptoms of disease
Source: BMC Microbiol. 2010 Mar 31;10:96. doi: 10.1186/1471-2180-10-96 (PMC2858740; doi:10.1186/1471-2180-10-96)

|                                                                                   | Isolate      | Phagetype | PFGEmodel |
|-----------------------------------------------------------------------------------|--------------|-----------|-----------|
| 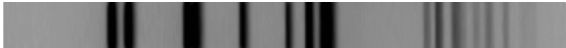   | 01 11M1 2249 | RDNC      | 327       |
| 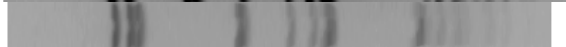  | 02 08F10 996 | 193       | 329       |
| 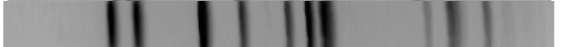  | 01 10F70 02  | 120       | 49        |
| 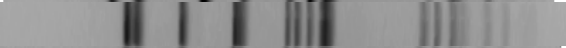  | 02 05R43 81  | 12        | 22        |
| 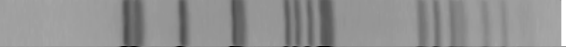  | 05 06H32 341 | 12        | 22        |
| 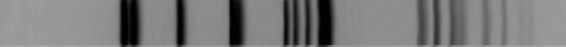  | 01 12F33 212 | 12        | 22        |
| 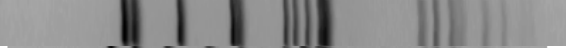  | 02 02F44 678 | 12        | 22        |
| 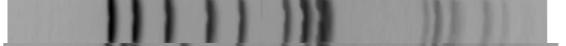  | 02 11F40 143 | RDNC      | 327       |
| 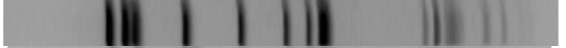  | 01 10H11 581 | 10        | 29        |
| 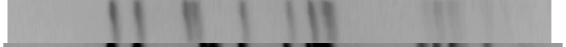  | 02 07M7 2344 | RDNC      | 61        |
| 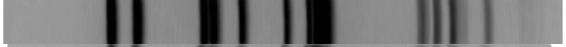  | 01 11H24 126 | 104       | 14        |
| 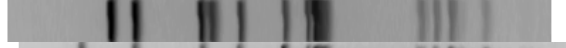  | 02 10H31 581 | 104       | 14        |
| 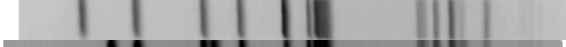  | 05 09R68 52  | 104       | 205       |
| 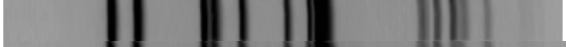  | 01 10R39 88  | 104a      | 14        |
| 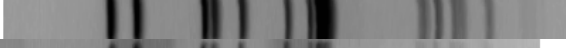  | 01 12F28 702 | 104       | 14        |
| 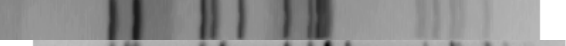  | 05 11R70 26  | 104       | 14        |
| 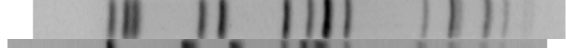  | 02 10F37 188 | 3         | 76        |
| 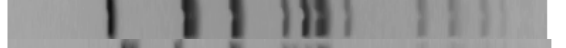  | 02 10M1 6322 | 170       | 195       |
| 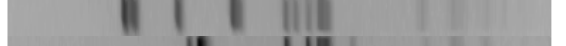  | 02 07T97 64  | 12        | 22        |
| 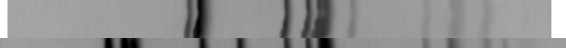  | 02 09H16 582 | 120       | 6         |
| 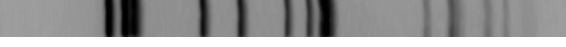 | 02 01H32 554 | 10        | 255       |

Supplement: Additional file 1 — PFGE profiles. Xba I PFGE profiles of all isolates [file 1471-2180-10-96-S1.PDF]
