# Supplementary material for: DNA microarray analysis of Salmonella serotype Typhimurium strains causing different symptoms of disease
Source: BMC Microbiol. 2010 Mar 31;10:96. doi: 10.1186/1471-2180-10-96 (PMC2858740; doi:10.1186/1471-2180-10-96)
Supplement: Additional file 2 — Typing results of all strains [file 1471-2180-10-96-S2.DOC]

| Isolate nr | MLVA profile STTR- | | | | | Resistance profile | PFGE profile | Sequence Type |
| --- | --- | --- | --- | --- | --- | --- | --- | --- |
|  | 9 | 5 | 6 | 10 | 3 |  |  |  |
| 0210F37188 | 4 | 15 | 8 | -1 | 0111 | susceptible | 76 | 19 |
| 0110H11581 | 2 | 12 | 20 | 11 | 0212 | susceptible | 29 | 376 |
| 0202F44678 | 4 | 17 | 10 | 7 | 0211 | susceptible | 22 | 19 |
| 0205R4381 | 4 | 13 | 11 | 7 | 0211 | susceptible | 22 | 19 |
| 0111H24126 | 3 | 12 | 16 | 23 | 0311 | AMP,CHL,FFN,SMX,SPT,STR,TET | 14 | 19 |
| 0210H31581 | 3 | 14 | 19 | 21 | 0311 | AMP,CHL,FFN,NAL,SMX,SPT,STR,TET | 14 | 19 |
| 0110F7002 | 4 | 13 | 10 | 7 | 0211 | AMP,AMC,CHL,SMX,SPT,STR,TET | 49 | 19 |
| 0209H16582 | 3 | 12 | 9 | -1 | 0211 | susceptible | 6 | 34 |
| 0211F40143 | 2 | 12 | 19 | 9 | 0212 | susceptible | 323 | 19 |
| 0201H32554 | 4 | 15 | 7 | -1 | 0111 | susceptible | 255 | 19 |
| 0112F33212 | 4 | 13 | 13 | 8 | 0211 | susceptible | 22 | 19 |
| 0207T9764 | 4 | 11 | 12 | -1 | 0211 | susceptible | 22 | 35 |
| 0112F28702 | 3 | 13 | 16 | 31 | 0311 | susceptible | 14 | 19 |
| 0110R3988 | 3 | 13 | 11 | 26 | 0311 | AMP,CHL,SMX,SPT,STR,TET | 14 | 19 |
| 0210M16322 | 3 | 15 | 14 | -1 | 0311 | SMX,STR,TET | 195 | 19 |
| 0208F10996 | 4 | 15 | 11 | 7 | 0211 | SMX,SPT,TET,TMP | 329 | 19 |
| 0111M12249 | 2 | 11 | 11 | 10 | 0212 | AMP,SMX,SPT,TET,TMP | 327 | 19 |
| 0207M72344 | 2 | 19 | 19 | 11 | 0212 | susceptible | 61 | 19 |
| 0506H32341 | 4 | 14 | 11 | 8 | 0211 | susceptible | 22 | 19 |
| 0509R6852 | 3 | 11 | 18 | 17 | 0311 | AMP,CHL,FFN,SMX,SPT,STR,TET | 205 | 19 |
| 0511R7026 | 3 | 14 | 14 | 23 | 0311 | susceptible | 14 | 19 |

AMC=amoxicillin-clavulanic acid, AMP=ampicillin, CHL=chloramphenicol, FFN=Florfenicol, NAL=nalidixic acid, SMX=Sulphamethoxazole, SPT=spectinomycin, STR=streptomycin, TET=tetracycline, TMP=trimethoprim
