# Supplementary material for: DNA microarray analysis of Salmonella serotype Typhimurium strains causing different symptoms of disease
Source: BMC Microbiol. 2010 Mar 31;10:96. doi: 10.1186/1471-2180-10-96 (PMC2858740; doi:10.1186/1471-2180-10-96)
Supplement: Additional file 3 — Microarray results of all markers. Markers are listed alphabetically within marker groups. A grey box indicates the marker being present and a white box indicates the marker being absent. [file 1471-2180-10-96-S3.PDF]

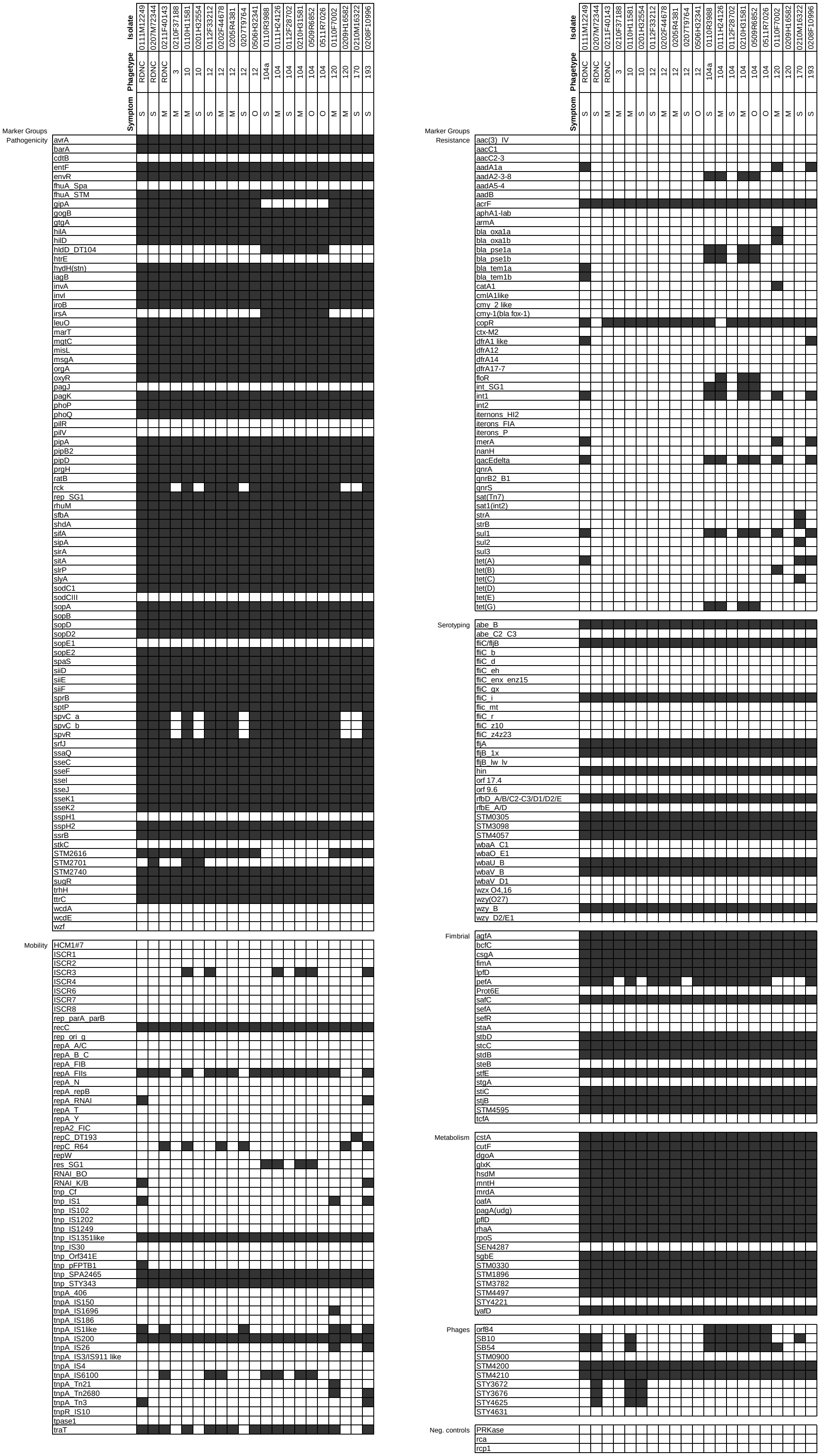

Isolate
0111M12249
0207M72344
0211F40143
0210F37188
0110H11581
0201H32554
0112F33212
0202F44678
0205R4381
0207T9764
0506H32341
0110R3988
0111H24126
0112F28702
0210H31581
0509R6852
0511R7026
0110F7002
0209H16582
0210M16322
0208F10996
Phagetype
RDNC RDNC RDNC 3 10 10 12 12 12 12 12 104a 104 104 104 104 104 120 120 170 193
Symptom
S S M M M S S M M S O S M S M O O M M S S
Marker Groups
Pathogenicity
avrA
barA
cdtB
entF
envR
fhuA_Spa
fhuA_STM
gipA
gogB
gtgA
hilA
hilD
hldD_DT104
htrE
hydH(stn)
iagB
invA
invI
iroB
irsA
leuO
marT
mgtC
misL
msgA
orgA
oxyR
pagJ
pagK
phoP
phoQ
pilR
pilV
pipA
pipB2
pipD
prgH
ratB
rck
rep_SG1
rhuM
sfbA
shdA
sifA
sipA
sirA
sitA
slrP
slyA
sodC1
sodCIII
sopA
sopB
sopD
sopD2
sopE1
sopE2
spaS
siiD
siiE
siiF
sprB
sptP
spvC_a
spvC_b
spvR
srfJ
ssaQ
sseC
sseF
sseI
sseJ
sseK1
sseK2
sspH1
sspH2
ssrB
stkC
STM2616
STM2701
STM2740
sugR
trhH
ttrC
wcdA
wcdE
wzf
Mobility
HCM1#7
ISCR1
ISCR2
ISCR3
ISCR4
ISCR6
ISCR7
ISCR8
rep_parA_parB
recC
rep_ori_g
repA_A/C
repA_B_C
repA_FIB
repA_FIIs
repA_N
repA_repB
repA_RNAI
repA_T
repA_Y
repA2_FIC
repC_DT193
repC_R64
repW
res_SG1
RNAI_BO
RNAI_K/B
tnp_Cf
tnp_IS1
tnp_IS102
tnp_IS1202
tnp_IS1249
tnp_IS1351like
tnp_IS30
tnp_Orf341E
tnp_pFPTB1
tnp_SPA2465
tnp_STY343
tnpA_406
tnpA_IS150
tnpA_IS1696
tnpA_IS186
tnpA_IS1like
tnpA_IS200
tnpA_IS26
tnpA_IS3/IS911 like
tnpA_IS4
tnpA_IS6100
tnpA_Tn21
tnpA_Tn2680
tnpA_Tn3
tnpR_IS10
tpase1
traT
Resistance
aac(3)_IV
aacC1
aacC2-3
aadA1a
aadA2-3-8
aadA5-4
aadB
acrF
aphA1-Iab
armA
bla_oxa1a
bla_oxa1b
bla_pse1a
bla_pse1b
bla_tem1a
bla_tem1b
catA1
cmlA1like
cmy_2 like
cmy-1(bla fox-1)
copR
ctx-M2
dfrA1 like
dfrA12
dfrA14
dfrA17-7
floR
int_SG1
int1
int2
iternons_HI2
iterons_FIA
iterons_P
merA
nanH
qacEdelta
qnrA
qnrB2_B1
qnrS
sat(Tn7)
sat1(int2)
strA
strB
sul1
sul2
sul3
tet(A)
tet(B)
tet(C)
tet(D)
tet(E)
tet(G)
Serotyping
abe_B
abe_C2_C3
fliC/fljB
fliC_b
fliC_d
fliC_eh
fliC_enx_enz15
fliC_gx
fliC_i
flic_mt
fliC_r
fliC_z10
fliC_z4z23
fljA
fljB_1x
fljB_lw_lv
hin
orf 17.4
orf 9.6
rfbD_A/B/C2-C3/D1/D2/E
rfbE_A/D
STM0305
STM3098
STM4057
wbaA_C1
wbaO_E1
wbaU_B
wbaV_B
wbaV_D1
wzx O4,16
wzy(O27)
wzy_B
wzy_D2/E1
Fimbrial
agfA
bcfC
csgA
fimA
lpfD
pefA
Prot6E
safC
sefA
sefR
staA
stbD
stcC
stdB
steB
stfE
stgA
stiC
stjB
STM4595
tcfA
Metabolism
cstA
cutF
dgoA
glxK
hsdM
mntH
mrdA
oafA
pagA(udg)
pflD
rhaA
rpoS
SEN4287
sgbE
STM0330
STM1896
STM3782
STM4497
STY4221
yafD
Phages
orf84
SB10
SB54
STM0900
STM4200
STM4210
STY3672
STY3676
STY4625
STY4631
Neg. controls
PRKase
rca
rcp1
